# Short and Locked Down: The Impact of COVID-19 on a Person With Dwarfism

**DOI:** 10.1177/12063312231181519

**Published:** 2023-07-14

**Authors:** Clare Harvey

**Affiliations:** 1 University of the Witwatersrand, Johannesburg, Gauteng, South Africa

**Keywords:** autoethnography, biopolitical power, COVID-19 pandemic, dwarfism, physical disability

## Abstract

Living with a visible physical disability—specifically dwarfism—brings situational, psychosocial, and cultural challenges. The COVID-19 pandemic, and its restrictions, amplifies these dwarfism-related complexities, exposing the politics of visibility and exclusion, as well as spatial injustices. This autoethnographic paper deliberates these heightened disabling encounters in their various contextual layers—physical, social, and psychological. Fundamentally, people with dwarfism have become further disabled and disadvantaged because of the pandemic’s psychosocial stresses, contextual traumas, and physical exclusions. The paper intimately addresses the embodied, psychological, cultural, and spatial inequalities short-statured individuals endure because of the pandemic. Drawing on theoretical models of disability, critical disability literature, geographies of disability, as well as the conceptual paradigm of biopolitical power, the paper begins to make sense of these experiences so that shifts may occur in different spaces. Arguably, the experiences of COVID-19 that are shared here are also applicable to people with other disabilities.

## Backdrop

In recent decades, disability has been enshrined within the framework and discourse of human rights and access, including in the United Nations Convention on the Rights of Persons with Disabilities (2006). Yet, despite these protections, disabled people continue to endure social marginalization, disablism, and spatial relegations. “The systematic exclusion of disabled perspectives has often allowed ableist policy and social norms to go unchecked or unchallenged” (Lund et al., 2020, p. 314). Most physical spaces are designed by (able-bodied) individuals with, and for, the average—not disabled—person in mind. How users experience environments may or may not correspond with the designer’s intentions (Heylighen & Strickfaden, 2012). Frequently, disabled people are left disempowered and dependent on others for help because of spatial inadequacies. The social model of disability considers how one is disabled because of the exclusionary societal environment—people are constructed as disabled because they do not fit into the cultural and physical backdrop (Barnes, 2012). Indeed, “bodies are exposed to the architectural performance of pathological orderings and norms” (Schillmeier, 2020, p. 13). These marginalizing experiences also emphasize the physical (impaired) nature of disability within the person because disabled people are reminded that they are “other.” The medical model of disability views disability as a biological, pathological problem that resides in a person’s body, which needs to be remediated or cured (Ryan, 2005). Space cannot be focused on without taking a geographical lens, specifically a geographies of disability view which covers “the socio-spatial experiences of disability” (Pritchard, 2021, p. 2). Thus, environments are both structural and socially produced with meaning in how they are used.

Arguably, noticeable disabilities—those visible to others—invite specific challenges, particularly experiences of disablism, within the physical, social, and psychological milieu. From a psychological perspective, disability can feel intolerable to think about and threatening to engage with for both disabled and able-bodied individuals. Kristeva (2010) states:

The disabled person opens a *narcissistic identity wound* in the person who is not disabled; he [or she] inflicts a threat of *physical or psychical death*, fear of collapse, and, beyond that, the anxiety of seeing the very *borders of the human species* explode. And so *the disabled person is inevitably exposed to a discrimination that cannot be shared*. (p. 251, emphases in original)

Thus, disabled people and their bodies constantly remind able-bodied people of that which they are trying to avoid or ignore (Wendell, 1997); it is terrifying to think one can become disabled. Coping with this intolerable terror may involve defending against these feelings by ridiculing the disabled ‘other’—staring, laughing, pointing, and so on—creating an experience of alienation and shame for disabled people within psychosocial spaces (see, Harvey & Long, 2020). A critical, psychoanalytic lens to disability—the feminist model—encompasses a personal and experiential perspective. This article takes a holistic approach to understanding disability, engaging with all the facets of this experience—medical, physical, social, cultural, and psychological. Indeed, the material and immaterial (feelings, lived experience, social inclusion, citizenship, etc.) cannot be separated; and sociocultural identity cannot be considered separate from the spatial environment (Heylighen & Strickfaden, 2012): “(O)ur embodiment in the world constructs physical and social interaction” (p. 182).

Short-statured individuals—people with dwarfism—form one of the most noticeable physically disabled groups. “Dwarfism is a dramatic, physically distinctive, and immediately identifiable condition” (Ablon, 1990, p. 880). I am one such individual. I have Achondroplasia, a common form of disproportionate dwarfism. My physical appearance precedes me with my large head, short arms and legs, and dominant dwarfism gait. I stand only 4 ft tall (120 cm) with a very short arm reach. I am also female, South African, an academic, and a clinical psychologist. Acceptable terminology for a person with dwarfism is highly contested (Pritchard, 2015)—with “dwarf,” “short-statured,” “little person,” and “person with restricted growth” all acceptable terms. Similarly, disability scholars vary in their views of language use. In this article, I use enabling language—disabled people—because I understand disability to be a social construct (Barnes, 2012). Yet, people with dwarfism also have a medical impairment in their physical complications associated with their short stature. Hence, I believe “dwarf” and “person with dwarfism” are both acceptable.

My disability-related experiences before the pandemic can be described as challenging at best—in the social, physical, as well as psychological domains. I am confronted with the politics of my physical disability on a daily basis. Certainly, I cannot write about space and its ability to regulate, limit, and control disabled people without considering how society and culture are based on power relations (Foucault, 1975). Indeed, “space is a set of power relations” (Yoltay, 2021, p. 587), and practices of domination and relegation are entrenched. The framework of biopolitics hangs over those who are “different” so as to uphold normalization within society. In this way, dominant rationalities, as well as boundaries, are established through which society is understood and can function (Campbell & Sitze, 2013). Furthermore, the biology of people are organized into a political existence (Mills, 2015). Being short in a tall world constantly reminds me that the built environment does not include me. I am often seen and related to through one lens—that of my disabled body—at the expense of relating to me in my entirety, made up of different identities. My visible disability is not always welcomed within social spaces. People point, stare, even laugh at me—I am ridiculed for my difference: the “unremitting, pathologising violence of the non-disabled gaze” (Hughes, 2009, p. 406). My otherness is perceived as a threat to the (able-bodied) normative social order and thus power is maintained by relegating me to the margins of society.

The coronavirus pandemic (COVID-19) and its specific restrictive lifestyle implementations—lock downs, face masks, perspex barriers, demarcated social, and physical distancing spaces—have greatly amplified the spatial, social, cultural, and psychological challenges for disabled people. Sharing space with the virus affects my everyday life, in ways that able-bodied people do not experience. As Shields et al. (2020) state, “staying six feet apart to avoid infection is just the tip of the lived experience of the pandemic, quarantine, and isolation” (p. 216). These complex, embodied experiences are explored in this article. Reference is made to the different models of disability, critical disability literature, geographies of disability, as well as the theoretical framework of biopolitical power, to begin to make sense of my disability-related experiences within a COVID-19 world.

The lived experience of people in different spaces has been explored from various perspectives and theoretical paradigms, including those of performance, embodiment, and bodily engagement (Devlieger & Strickfaden, 2012; Fitzsimons, 2012; Nijs & Daems, 2012). While these are important contributions, the foci of these papers are not on the experiences of physically disabled people.

While some authors have reflected on various space adjustments and regulations as a consequence of the pandemic, these too are not from the perspective of disabled people—specifically those with dwarfism (e.g., Shields et al., 2020; Van Assche et al., 2020). Authors have begun to address disabled people’s experiences of the pandemic. For example, Ned et al. (2020), McKinney et al. (2021), as well as Hearn et al. (2022) have considered health care of disabled people within the context of the pandemic. These papers are valuable contributions, yet these too are not specifically related to the embodied experiences of physically disabled people within various spaces. Taking it further, Kruse (2003) reminds us of the paucity in research focusing on the social aspects of dwarfism, and Pritchard (2021) argues for a greater geographical, spatial focus in understanding experiences of the disabling built environment.

Furthermore, “inclusion . . . is not an end-state but rather something that must be constantly performed” (Fallov & Birk, 2020, p. 536). With the continually changing backdrop because of the pandemic, the process of inclusion needs to be constantly revisited—something this paper intends to do. The processes of the pandemic “have the power to disassemble as well as reassemble living societies” (Schillmeier, 2020, p. 10). Arguably, this reassembling can only be meaningful if disabled people’s voices are also heard.

## Autoethnography as Method

The article’s method is autoethnographic—a subjective qualitative research technique and style of writing exploring an “individual’s unique life experiences in relationship to social and cultural institutions” (Custer, 2014, p. 1). Qualitative research is useful here because it questions assumptions and adds knowledge with the belief that positive changes can be made for people (Thorne, 2011). Autoethnography falls within the phenomenological research paradigm, reflecting on people’s experiences. It is a form of social inquiry with individuals within a context (Larson, 1997). Hence, weaving together the inner with the outer realities, the personal with the public, the emotional with the intellectual (Lourens, 2020).

Autoethnography is a demanding yet powerful technique in that it requires the researcher to live “consciously, emotionally, reflexively . . . that we observe ourselves observing, that we interrogate what we think and believe, and that we challenge our own assumptions” (Jones et al., 2013, p. 10). It is a method of narrating one’s story from the position of knower and discoverer (Richards, 2013). Autoethnography requires a vulnerability from the researcher so that their emotional, embodied experiences can be shared (Raab, 2013)—and herein, the researcher and reader may be transformed, and a change within the greater context may be ignited. Thus, autoethnography explores, challenges, and undoes the established ways of relating within different spaces. The method has proven successful in allowing “other” (minority and difference) experiences to be appreciated and understood within the relevant socio-political context—an aim of this article (Harvey & Kotze, 2022). Arguably, it is a particularly effective methodological approach for communicating disabled embodiment—untangling “the unspoken intricacies and interrelationships between a physical body that is disabled and a world that is not” (Lourens, 2020, p. 5).

Specifically, the study has taken an analytic autoethnography approach—data provide insight into and are used to “develop, refine and extend theoretical understanding” of social phenomena (Anderson, 2006; Le Roux, 2017, p. 201; Pace, 2012).

I write parts of my story here—those chapters that disclose my experiences of being short and locked down because of the pandemic. Through critically reading, understanding, and contextualizing these experiences in relation to theory (Le Roux, 2017)—specifically disability studies, geographies of disability, and biopolitics—I construct and reconstruct my experiences, and uncover the taken-for-granted hegemonic practices of various spaces. In this way, the data are interpreted and synthesized. An overarching focus has been a striving for an equal balance of raw data and academic discourse—thus, promoting rigorous analysis (Fine, 1998). Furthermore, Le Roux (2017) suggests autoethnographic studies need to be grounded in theoretical concepts to anchor, and bring rigor to, the study. This study aims for rigor in making sense of the narrative within the exiting literature, thus contributing to the discourse and academic discipline. A process of member checking (ibid), through the thorough review process of three different, independent reviewers, as well as the Editor of this journal is another way in which this article strives for rigor.

At times this was a vulnerable, painful approach to research—an expected aspect of autoethnography—so as to uncover and make sense of these experiences. Custer (2014) describes this research engagement and embodied writing as “courage and clarity of purpose” (p. 4). The output text, put down here, reveals the facets of the disabled self interacting with others within various spaces (Spry, 2001). Consequently, some of the power relationships within different contexts may be destabilized—a function of autoethnography (Richards, 2013).

## Short and Locked Down

With the pandemic came many restrictions, changing the social landscape across different spaces. These include ongoing strict lockdowns and social distancing policies, new social norms in the (legalized) wearing of face masks, recordings of people’s temperatures before entering buildings, and the ever-present requirement of sanitizing. While most of these are understandable, sensible prescriptions, they significantly make life with a disability that much more complex; highlighting and exacerbating already existent multiple layers of inequality, disadvantage, and marginalization. These restrictions and practices intersect with “established institutional and systemic forms of oppression, creating additional challenges and concerns for people from historically marginalised groups”—including disabled people (Lund et al., 2020, p. 313).

In 2020, South Africa legalized the wearing of face masks in all public settings. While I do not mind wearing a mask to protect myself and others against potential infection, it has negatively impacted my social and psychological worlds. Shields et al. (2020) state that masks have “changed our voices” (p. 218)—in fact, in many instances my mask has silenced me, relegating me to the periphery of society. Wearing a face mask creates an experience of greater disability (both spatially and psychosocially) for those with dwarfism. Conversing through masks with people much taller than me has created a strained experience in communication and connection. Pre-COVID-19, I needed to talk louder than usual when standing and communicating with average height people—my voice needed to travel the upward distance. The requirement of wearing a mask has restricted my communication, and I experience others as not being able to sufficiently hear me—ableism, and more specifically heightism, leaves me experiencing discrimination (Pritchard, 2021). Historically, reading the other’s lips in noisy environments has helped me “hear” them; yet lips are no longer visible in COVID-19 times. A disability lens of distance is apparent; and a height separation and sense of alienation dominates my engagements. “Our sense of being in the world and attachment to place arises from engagement with both the human and nonhuman (material and social) aspects of place” (Sims et al., 2009, p. 306). Arguably, this isolating experience would also be applicable for other forms of disability, notably those individuals that are hearing impaired. These COVID-19 restrictions negatively impact disabled people in social spaces. While the social model of disability has been critiqued for largely ignoring individual, personal, bodily, and emotional experiences of disability (Shakespeare, 2006), it is useful here in understanding my current experiences because of COVID-19. The world that I encounter is experienced as restricting, causing me to re-confront my disability, increasing my feelings of otherness. My experiences also hold a psychic lens—I am made to feel alienated and different, igniting various emotions within me, including anger and sadness. Thus, a complex, nuanced understanding of disability is necessary. A synthesis of the psychoanalytic approach to disability with the social and medical models is useful (Goodley, 2011)—incorporating psychic, relational, social, geographical, physical, and medical perspectives of disabled experiences: an embodied model of disability, creating an understanding of disability that is encompassing for disabled people.

The immaterial challenges of disability (the lived experiences, emotional engagements, social encounters) can be confounded by the material, physical restrictions of space, creating a complex multilayered interaction (Heylighen & Strickfaden, 2012). The pre-existing discriminatory, unaccommodating public physical spaces and spatial barriers that people with dwarfism endure (Pritchard, 2021) can be more disabling because of the pandemic. Perspex screens and barrier tape to ensure physical distance aggravates prevailing disability-related communication and accessibility difficulties. My local pharmacy is an unpleasant, disabling environment which I need to interact with on a monthly basis. My dwarfism brings chronic physical pain which is managed through various practices, including taking medication. Relying on medication is a difficult aspect of my disability—both psychically and bodily. I am reminded that my body is the site of my disability; “the body is key to experiencing” (Heylighen & Strickfaden, 2012, p. 182). Thus, a medical model of disability experience is apparent—my disability is pathological, something needs to be remediated within my physical make-up through medicine (Ryan, 2005). The high pharmacy counters pre-COVID-19 created an alienating, othering experience for me—a geographical lens to disability where space reproduces disabled people’s exclusion from the environment (Kitchin, 1998). Adding masks, perspex boards, and barrier tape to keep me at an acceptable physical distance means I need to shout my order of medication. I feel exposed, vulnerable, and more disabled in these complex interactions of spatiality, embodiment, boundaries, and abilities. This unwanted social attention highlights the intertwining of the social-spatial (Pritchard, 2021). I am disabled and othered by the physical restrictions because of the pandemic, thus the social model of disability is also applicable to these experiences (Barnes, 2012). Furthermore, I feel out of place, I do not belong because material objects do not support my interactions. Indeed “access to space creates a sense of citizenship” (Heylighen & Strickfaden, 2012, p. 182)—am I not a citizen in my own society? Undoubtedly, struggling to access basic aspects needed for life “embodies an attack on dignity” (Sadiki et al., 2021, p. 9). Taking it further, these powerful effects of the normalized, able-bodied, taken-for-granted relations of bodies, spaces, and cultures are ingrained to the point of disabling anyone who differs from the norm (Schillmeier, 2020). The broad societal structures impose compulsory able-bodiedness (Harvey & Kotze, 2022). Thus, disabled people are socially and politically excluded. The pharmacy’s COVID-19-specific structurally embedded oppression of disabled people leaves me feeling individualized and alienated. Thus, an emotional, psychological understanding of disability is also pertinent in these experiences. These complex spatial configurations dictate my accessibility to life around me (Rieger et al., 2019) and are not only reserved to the space of my local pharmacy.

Living in chronic pain also means regular hospital visits for X-rays, cortisone injections, as well as a minor operation since the start of the pandemic. Paradoxically, even in this medically framed space I am excluded. I am confronted with the medical model of disability because I am reminded that I live in a body that is pathological and dependent on (able-bodied) others. However, a social model of disability practice is also evident since the hospital surroundings make me feel othered. The space is not made with disabled, specifically short-statured, people in mind. The built environment caters for “the able-bodied, average-sized person and thus public spaces are oppressive and exclusionary for people who do not fit these standards” (Pritchard, 2015, p. 1). Spaces are “staturized” (Kruse, 2010). There is a fixed thermometer reader on the entrance wall to the hospital—it is too high to read my temperature and so I am stopped and not allowed in while security ponders what they should do about me. I am never allowed to “forget” my disability, and merely exist in space. Being disabled in an able-bodied world often makes disabled people feel like misfits, causing a need to outperform and over-achieve so as to feel “accepted” by able-bodied people, going so far as to try and present as not disabled—to supercrip (Garland-Thomson, 2011; Harvey, 2015). I do not complain that the hospital does not have a portable thermometer, rather I excuse them for their oversight because I am the one “causing a problem.” In turn I am left with an array of difficult feelings because of my embodiment—shame, embarrassment, sadness, frustration, and exhaustion. “Embodied space is the location where human experience and consciousness take on material and spatial form” (Low, 2003, p. 10). In addition, my hospital experience can be understood within the context of the politics of performance, which “requires” those that are “other” to adhere to the power structures of society. Power is established through the normalization of dominant rationalities (Mills, 2015)—and disabled people are made to perform a normative type of existence so as to re-enact normativity. Intimately linked to the politics of performance is the politics of affect. Performing as a “good” citizen who does not question the status quo of the discriminatory spaces around me means that my emotions function to “mediate the relationship between the psychic and the social, and between the individual and the collective” (Ahmed, 2004, p. 119). Hence, power relations between the dominant, “normal” group and the minority, “other” group are upheld. The emotional labor that accompanies disability is exhausting (Harvey, 2017).

Living with a disabled body is also challenging in other public, able-bodied spaces. Having dwarfism means I can only reach certain items in shops—my very restricted arm reach often hinders me from acquiring what I need or want in a shop. Such barriers “cement harmful stereotypes about . . . the dependency of people with disabilities” (Sadiki et al., 2021, p. 9). (Reluctantly) asking for assistance from (able-bodied) members of the public to reach items down for me has been met with a noticeably greater hesitancy in these pandemic times. The refiguration of social (boundaried) space because of the pandemic and the idea that “bodies are suspect because they are involuntary toxic” creates a distanced, isolated society (Shields et al., 2020, p. 218). We are not meant to touch others or that which others have touched. The need to socially distance and avoid human contact creates an experience of isolation for everyone; arguably more so for disabled people. Schillmeier (2020) points out that COVID-19 “makes us experience the vulnerability of public life” (p. 2). Not only am I met with the threats from the virus, but I am also faced with my own dependency needs and the fact that I have to make myself vulnerable so as to be assisted. My disproportionate difference and inability scream at me when I need to rely on the physicalness of others because of the inadequacy of my own body. Indeed, there is a close relationship between the emotional and physical landscapes (Schillmeier & Domènech, 2009). Being reminded of my physical limitations within a disabling physical and social world are more destressing because of these COVID-19 restrictions. Again, an integrated, embodied model of disability—encompassing the medical, social, geographical, and psychic—is helpful in making sense of my experiences. I am also challenged by the conflict of wanting to be fiercely independent and thus not disabled, yet the space around me does not allow this. As Lourens (2020) states, disabled people internalize the able-bodied oppressive messages that showing vulnerability and dependency relegates one to the margins of society. How do I embrace an integrated self? How do I own those parts of myself that I am both proud and ashamed of in a world in which disabled people are (only) respected when they “transcend” their disability (Harvey, 2015)? As a marginalized “other,” I am called upon to prove my worth; to perform able-bodiedness (Ahmed, 2009). I do not question or complain to others when they refuse to reach something for me in the supermarket. Instead, I pretend it is not an issue. Once again, I “adhere” to the power structures of society—that of able-bodiedness. I “behave” and do not confront the power relations between the dominant (not disabled) group and the minority (disabled) group. I am reminded that we all inhabit a world in which we do not all belong (Garland-Thomson, 2011).

The strain on people’s mental health because of the COVID-19 pandemic and the accompanying lifestyle changes is becoming more apparent. Reports of feelings of loneliness, marginalization, anxiety, among others, are all too real for many (Fiorillo & Gorwood, 2020). Yet, several of these experiences have been by my side most of my life because of my disability. Living through the pandemic has exacerbated these personal, emotional experiences. Outings to shops have become anxiety-provoking, not (only) because I am worried about contracting COVID-19, but also because others are not so willing to assist me when necessary. I am met with the “affective atmospheres of unwelcome” (Fontaine, 2021, p. 1). Certain bodies are not accepted in certain spaces. I have become physically and psychologically exhausted trying to fit in to the world around me. According to Ahmed (2015), “emotions show us how power shapes the very surface of bodies as well as worlds” (p. 12). Thus my affects, my experiences of my body within itself and in space, can be understood as politically sanctioned.

Furthermore, one’s sense of belonging is directly correlated with the connection between people and place (Abdollahyan & Mohammadi, 2020). To counteract cultural and psychosocial ableism I consciously attempt to defuse others’ (negative, even shocked and rejecting) responses to me and my visible disability. Certain disabilities in another—particularly dwarfism with its novel disproportionate appearance—is perceived as curious, strange, and unfamiliar by able-bodied people (Harvey, 2020). Within social spaces, I smile and warmly interact in an attempt to collapse some of the negative preconceptions the other may hold toward me and my disability. Psychologically, this is akin to disarming others’ defenses—they are forced to re-evaluate their own insecurities about disability. These political deviant acts of displaying a comfort with my own disability act to “hide” my own vulnerabilities and difference. Arguably, encountering disability in a person ignites discomfort in the (able-bodied) other because they are reminded of their own body’s vulnerability, fallibility, and the fact that they too may have been, or even worse, may still become disabled (ibid). Moreover, “expectations to perform specific invocations of marginalised identities produce the very surfaces of our bodies as different . . . producing an intense awareness of the cause of discomfort and tension we become to those who are not ‘other’” (Harvey & Kotze, 2022, p. 12). My body is a “sore point” for (able-bodied) others because it produces discomfort and tension in them (Ahmed, 2009, p. 51). Hence, in various social spaces, I try to make the other feel at ease so as to connect with them, and to hopefully not experience rejection. The current pandemic’s restrictions greatly inhibit this possibility, resulting in a loss of agency in relating as an “other” individual in the world, producing a sense of disempowering exclusion. Wearing a mask has gravely affected my social interactions because I cannot smile and warmly approach those around me. Awkwardly, I find myself winking at others in an attempt to relate and lessen their discomfort—an action that does not always have the desired effect of connection. Bantjes et al. (2019) write about how the inclusion of disabled people in certain spaces is reliant on disabled people’s enduring performances of cheerfulness, so as not to scare able-bodied people. I have been robbed of my practice of smiling cheerfully so as to make those who are able-bodied feel as comfortable as possible with me and my otherness. The emotional labor of being different is apparent. Indeed, “difference . . . invokes a perpetual interrogation of self in relation to the politically dominant ideal in society” (Harvey & Kotze, 2022, p. 9). Consequently, the power relationship with the “other” as marginalized is maintained, and the discomfort of able-bodied people is soothed (Ahmed, 2009). Furthermore, “while physical distance may be needed to slow the virus, social proximity is critical to absorb its impact” (Van Assche et al., 2020, p. 233). Yet, more than ever before, our social practices and affective relations within cultural spaces are mediated (Schillmeier, 2020). A new social performativity results (Shields et al., 2020)—negatively affecting people’s mental health. Arguably, as shown in this article, the disabled person’s experience of the pandemic’s restrictions in various spaces cause greater stress than that which is experienced by the dominant, normative, not disabled population.

Interestingly, this new COVID-19 world of working from home over zoom and other online platforms because of the need to social distance and isolate has centered my disability once more. These online spaces of connection and exchange ignite anxieties within me since I cannot show my whole physical, disabled self to those who I have never met. Paradoxically, it feels unknown and awkward for me that the other cannot necessarily see a prominent part of my identity. My disability usually precedes me and my encounters with others. I am left wondering who am I without my visible disability? On occasion I feel a need to perform my disability in these online spaces. Paradoxically, I (consciously) bring the politics of performance into the space—although this political agenda has been thrust upon me so many times that it has become a part of me. In these ever-present online spaces, I find myself performing my disability—I ensure my short, disproportionate arms are visible on the screen. Since power structures are ingrained within the fabric of society through the normalization of dominant rationalities (Mills, 2015), disabled people can feel pressurized to perform as able-bodied. Society calls me to perform certain expected aspects of my marginalized identity. “The neoliberal performance standards and discourse around disability automatically position them [disabled people] as misfits. . .and create disabling conditions within which they must perform and measure up” (Waterfield et al., 2018, p. 344). Compulsory able-bodiedness as the dominant framework of society burdens the “other” to make their difference apparent (Harvey & Kotze, 2022). The colleague on the screen, who occupies a powerful normative position—not disabled—evokes “deviant” responses in me—the othered, disabled self. In this way, the other person’s (and society’s) unfounded othering practice is justified (ibid). These acts alert me to how much discomfort my body causes (able-bodied) others, leaving me with feelings of exhaustion, anxiety, sadness, and anger. Hence, the politics of affect (Ahmed, 2015) are at play here too—my body is centered in these spaces, and I am confronted with the ambivalence between my internal, emotive experiences and my external, performative efforts.

## In Closing

The article has highlighted how the vulnerabilities and imbalances exposed by the COVID-19 pandemic reproduce and intensify existing disability-related embodied and spatial inequalities. The pandemic has deepened existing exclusions along othering lines in society.

Sharing my story has revealed how the pandemic locks me down in several ways. I am made to feel physically and psychologically small. I am looked down on as “other” because of my dwarfism. In addition, I feel locked down and alienated in my experiences of social otherness and physical alienation in spaces that are made by, and for, able-bodied people. The complex relationship between my visibly different body and my environment has intensified because of COVID-19. The various barriers—mask-wearing, physical barriers, social distancing, the world of online work—that the pandemic has brought are relational, embodied, material, and structural in nature. I have shared how, because of these barriers, I experience an intimate intertwining of the real functional limitations of my body—the relational, limiting and emotive aspects of disablism, alienation, and marginalization—as well as the lack of disability-friendly spaces. Thus, the pandemic’s limitations in the contexts around me have greatly exacerbated my disability-related encounters. I am constantly unmoored in my sense of self, security, and belonging because of how I am limited in what I can access, and who I can be. Some sense of these experiences has been made by drawing on critical disability literature, as well as on the medical, social, geography, and psychoanalytic models of disability—an embodied emotional model of disability. In addition, the paradigm of the biopolitical has been helpful—I have shared my visibly disabled lived reality of power (including the politics of affect and performance) in these COVID-19 times and spaces.

I hope that the article has created new understandings of exclusionary spatiality, bodily ability, and embodiment. Perhaps telling my disabled experience of the COVID-19 pandemic and questioning my sense of belonging to “home” will stimulate positive change in physical and psychosocial spaces. Disabled people should be included in designing and adapting spaces to create more encompassing environmental designs. Physical spaces should not communicate that able-bodiedness, and specifically tallness, is revered. Low counters need to be installed in public spaces such as pharmacies; access to hospitals should cater for all heights in people by having portable thermometers. In addition, society needs to be educated on difference. Meaningful, nonbinary policy and cultural shifts toward disability inclusion need to be made, encompassing the nuanced, intertwined disability experience. Society must acknowledge and appreciate the “plurality of beings” (Kristeva, 2003, p. 31). Spatial barriers can lead to social barriers (Pritchard, 2021), and thus if the built environment was more disability-friendly, society would be educated and encouraged to be more inclusionary in their attitudes and interactions with disabled people. Hence, I may not feel so alienated when encountering those around me, lessening the need to perform.

Being a clinical psychologist, I access my own therapy to help deal with the various encounters and experiences I have shared in this article. I also make sense of my experiences through some of my academic writing—including here. Personal narratives—particularly in the area of disability studies—are important ways in which we learn about ourselves and others (Garland-Thomson, 2005). People with dwarfism, and the greater community of disabled people, as with able-bodied people, should be encouraged to access mental health services to assist in processing our experiences, particularly in times of change and resulting stress, which COVID-19 has brought.

Finally, an embodied perspective of disability should be claimed, while also acknowledging the spatial exclusions, and bodily limitations of the disability experience. Thus, alternate understandings of disability-space relationships can be created: a nuanced, holistic, embodied model of disability—integrating the bodily, social, spatial, and psychological experiences and understandings of disability.
